# Representativeness of a national, probability-based panel survey of COVID-19 isolation practices—United States, 2020–2022

**DOI:** 10.3389/fepid.2024.1379256

**Published:** 2024-04-26

**Authors:** Holly H. Matulewicz, Divya Vohra, Willow Crawford-Crudell, John E. Oeltmann, Patrick K. Moonan, Melanie M. Taylor, Chandra Couzens, Andy Weiss

**Affiliations:** ^1^ Mathematica, Cambridge, MA, United States; ^2^ U.S. Centers for Disease Control and Prevention, COVID-19 Response Team, Atlanta, GA, United States

**Keywords:** representativeness, panel survey, validity, isolation, COVID-19, SARS-CoV-2

## Abstract

The U.S. Centers for Disease Control and Prevention (CDC) received surveillance data on how many people tested positive for SARS-CoV-2, but there was little information about what individuals did to mitigate transmission. To fill the information gap, we conducted an online, probability-based survey among a nationally representative panel of adults living in the United States to better understand the behaviors of individuals following a positive SARS-CoV-2 test result. Given the low response rates commonly associated with panel surveys, we assessed how well the survey data aligned with CDC surveillance data from March, 2020 to March, 2022. We used CDC surveillance data to calculate monthly aggregated COVID-19 case counts and compared these to monthly COVID-19 case counts captured by our survey during the same period. We found high correlation between our overall survey data estimates and monthly case counts reported to the CDC during the analytic period (*r*: +0.94; *p* < 0.05). When stratified according to demographic characteristics, correlations remained high. These correlations strengthened our confidence that the panel survey participants were reflective of the cases reported to CDC and demonstrated the potential value of panel surveys to inform decision making.

## Background

Local and state health departments report limited public health data to the U.S. Centers for Disease Control and Prevention (CDC) to monitor the number of people who tested positive for SARS-CoV-2 (1–3). Although routine case-based surveillance can enumerate the people notified by public health programs, as a nation, we knew much less about the actions of individuals who tested positive or received an exposure notification. To fill the information gap, we conducted an online, probability-based survey among a nationally representative panel of adults living in the United States to better understand the experiences and behaviors of individuals following a positive SARS-CoV-2 test result. This survey was designed to provide information and fill a gap in public health knowledge that could not be achieved through routine programmatic and surveillance data. Although the potential contributions of the survey were many, there were concerns about the panel survey design. These included the representativeness of survey participants relative to the population of the United States, given low response rates are often associated with population-based panel surveys, and the potential for recall bias that results from reflecting on life experiences more than a year past. The within-panel completion rate for the survey was strong (70%). The overall response rate was 4% and was computed in accordance with American Association of Public Opinion Research standards (4).^1^ Low response rates and non-response bias do not always directly correlate (5, 6), but low rates may raise concerns about the representativeness of the findings. We were concerned that potential sample bias and recall error could threaten the value of our findings. Herein we examine how well the panel responses (7) aligned with public health data reported to CDC (1). To assess sample bias, it is ideal, though usually not possible, to compare the characteristics of the survey respondents with a gold standard, in the same period, and on the same measures of interest. For this analysis, we had a unique opportunity to correlate and validate our survey data against the gold standard for COVID-19 programmatic and surveillance data collected and maintained by CDC.

## Methods

In January 2020, CDC began collecting COVID-19 case reports from public health jurisdictions to track trends of positive case counts and fatalities (8) by state, and by local jurisdictions such as county (2, 9–11). COVID-19 case-based reporting includes individual demographic characteristics such as age, sex, and race/ethnicity (1). CDC released weekly aggregated case-based COVID-19 surveillance and mortality data beginning in March 2020 (12). We used a probability-based panel survey of a nationally representative sample to understand the actions of people who self-reported positive SARS-CoV-2 test results (7). Detailed survey, sampling, and weighting methodology is available in the supplemental material. Briefly, we drew the sample from the Ipsos KnowledgePanel®, a probability-based, web-based panel that provides a representative sampling frame for all noninstitutionalized adults who resided in the United States (13). An address-based recruitment method based on the US Postal Service's Delivery Sequence File, stratified random sampling, and *a priori* weighting ensured that the geodemographic composition was comparable with the US adult population (7). We sought to compare monthly COVID-19 case counts based on our survey data with CDC's case-based, line-level surveillance data to answer the following questions:

1. How well did the case-based survey data align with CDC data of the number of reports of all adults (aged 18 years or older) who tested positive for SARS-CoV-2?
2. How well did the case-based survey data align with CDC data of the number of reports of all adults who tested positive for SARS-CoV-2 by select demographic characteristics?

We obtained aggregated, publicly available data from CDC (14). We calculated monthly aggregated case counts from March 2020, the first month for which the aggregated data are available, through March 2022 by summing weekly counts of all adults reported to provide comparability to the survey responses of adults who participated. We also subtracted monthly aggregated case-fatality counts from the surveillance data because the survey results excluded fatalities. We generated epidemiologic curves of both the survey data and CDC surveillance data to visualize the distribution of COVID-19 cases over time estimated by each data source and stratified by age, sex, and race/ethnicity. We then calculated Pearson's correlation coefficients (*r*) and associated *p*-values, comparing the surveillance data and weighted survey case counts. We calculated these correlation coefficients for each age, sex, and race/ethnicity group and for all adults age 18 and older.

### Findings

Here, we provide results from the analysis that compared the survey and surveillance data by research question.

#### Question 1

How well did the case-based survey data align with CDC data of the number of reports of all adults who tested positive for SARS-CoV-2?

**Figure 1:**
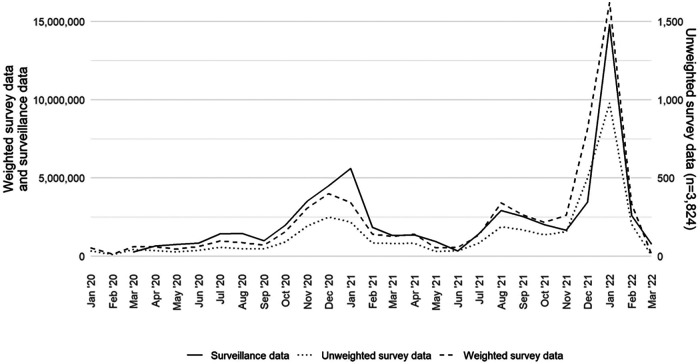
Monthly COVID-19 case counts of adults aged 18 years or older in the United States by data source (surveillance, weighted survey, and unweighted survey), excluding fatalities, January 2020–March 2022.

Figure 1 presents a comparison of survey-based monthly case counts, both weighted and unweighted, and surveillance-based monthly case counts reported to CDC from January 2020 to March 2022.

The weighted survey case counts mirror the temporal trends of the epidemiologic curve, as represented by the surveillance data. There was a strong correlation coefficient between the weighted survey and surveillance data (*r*: +0.94; *p* < 0.05). Although they are on a different scale, the unweighted survey cases also follow the epidemiologic curve. We compared the weighted survey data against the surveillance data with and without fatality counts included. From March 2020 to March 2022, fatality counts comprised 1.4% of the overall case counts in the surveillance data. The results for including and excluding fatality counts were the same at two decimal places and strongly correlated (*r*: +0.94; *p *< 0.05).

#### Question 2

**Figure 2:**
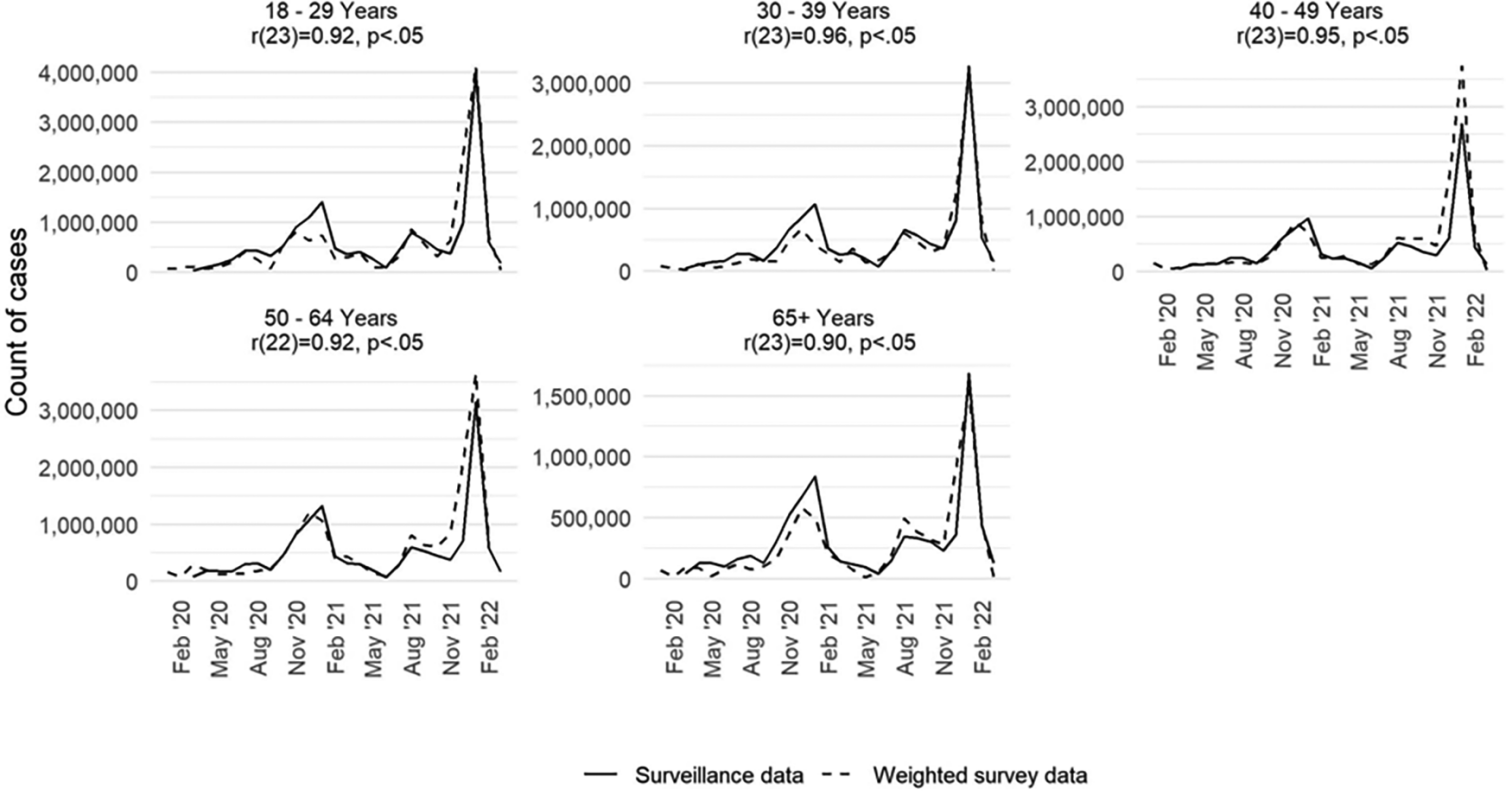
Monthly COVID-19 case counts in the United States by age group and data source (surveillance and weighted survey), excluding fatalities, January 2020–March 2022.

How well did the case-based survey data align with CDC data of the number of reports of all adults who tested positive for SARS-CoV-2 by select demographic characteristics? We recreated the epidemiologic curve with both data sets across five age groups (18–29, 30–39, 40–49, 50–64, 65 years and older). We found high correlation by age group between the survey and surveillance data for each group (Figure 2). For each age group, we note that the peaks for the survey and surveillance cases happen within 1 month of each other. The age group with the lowest correlation coefficient between the survey and surveillance data is the 65 years and older age group (*r*: +0.90; *p* < 0.05). The age group with the highest correlation coefficient was the 30–39 age group (*r*: +0.96; *p* < 0.05).

Finally, we assessed how well the survey data estimated the epidemiologic curve by sex and racial and ethnic groups. Findings from this analysis are available in Supplementary Material Figures S1 and S2. For sex (Supplementary Material Figure S1), we saw high levels of correlation, mirroring the findings shown for the population overall. For race and ethnicity (Supplementary Material Figure S2), it was not feasible to conduct a one-to-one comparison across the two data sources. Some of race/ethnicity classifications in the two datasets were not comparable and there was a high degree of missingness (35.4%) for the race/ethnicity variable in the CDC case-reports. Nonetheless, we found that the surveillance and survey data had statistically significant correlation coefficients for the following race/ethnicity groups: Hispanic; Black, non-Hispanic; White, non-Hispanic; and Asian or Pacific Islander, non-Hispanic. However, due to lower survey counts of people who identified as American Indian or Alaskan Native, non-Hispanic, we cannot draw conclusions on the relationship between the surveillance and survey data for people in this group. Although we found high correlation coefficients for most of the race/ethnicity groups, these results are complicated by the aggregated surveillance data not reporting a category for two or more races, an option that is available in the survey data.

## Discussion

This panel survey represented an opportunity to collect meaningful information to guide pandemic response, by capturing common behaviors in response to a COVID-19 diagnosis. However, panel survey results are sometimes devalued on the basis of low response rates. This study suggests that despite low overall response rates, the information gained from the survey may be meaningfully representative. Few surveys have the opportunity to compare their findings against surveillance records for the same population, in the same period, and on the same measures of interest. This survey presented a unique opportunity to assess the validity of survey data by comparing against a gold standard—case-based data reported to CDC during the analytic period. This comparison served as a validation that the survey data collected mirrored the U.S. adult population of COVID-19 cases overall and by age group. We observed a strong correlation between COVID-19 case counts generated by the survey and those reported by the CDC. This correlation strengthens confidence that self-reported SARS-COV-2 test results in our survey are reflective of the cases reported to the CDC during that same time period. Thus, the estimates generated by this survey may fill information gaps to better understand the experiences and behaviors of cases and contacts across the pandemic (7). The survey data might be particularly valuable for creating population estimates and facilitating analysis of these data by different demographic characteristics, such as age or race, which are subject to high rates of missingness in surveillance data.

This analysis has some limitations. Each data set might not reflect the entirety of the population of interest. For example, the panel survey does not include some segments of the U.S. population, people with language or literacy barriers that preclude participation in English or Spanish, those residing in congregate settings that were hit hard by COVID-19 (e.g., nursing homes, assisted living centers, and correctional facilities), and those experiencing homelessness. Conversely, CDC case-based data does not include people whose positive test results were not reported to public health officials, such as those who used at-home tests. In addition, although the completion rate among sampled panel members was high, the response rate for this survey was low, as is common with most panel surveys (15).

Despite these potential limitations, the panel survey provided a valuable approach and method to quickly estimate the proportion of people who isolated or quarantined for COVID-19, which did not previously exist (7). For example, although reporting confirmed cases was mandatory during the earlier days of the pandemic, maintaining this requirement was difficult when home-testing kits became available. Recent estimates suggest as many as 12 million adults had results exclusively from home-based tests during the analytic period (16). These results suggested that during the later days of the pandemic, up to 18% of people who reported being a case tested themselves and would not have been counted in the CDC case-based, line-listed surveillance data. These findings also provide important insight on the value and potential quality of probability-based panel surveys. This may be especially valuable when the new data can help inform planning (17), such as in public health emergencies like the COVID-19 pandemic, when researchers require more complete demographic data than surveillance sources might provide. It is important to note that a low response rate alone does not mean the data quality is poor (18, 19). The results from our analysis provide supporting evidence that probability-based panel surveys, when created with scientific rigor and deployed successfully, can provide a valid mechanism to collect data from the U.S. adult population that serve to generate national estimates on topics of interest with a high degree of accuracy.

## Data Availability

The raw data supporting the conclusions of this article will be made available by the authors, without undue reservation.

## Footnotes

^1^The response rate computation is based on the following: a random sample of 22,514 panel members was drawn from Ipsos’ KnowledgePanel®. A total of 15,923 participants (excluding breakoffs) responded to the invitation, and 9,269 qualified for the survey, yielding a final stage completion rate of 70.7% and a qualification rate of 58.2%. The recruitment rate for this study, as reported by (13) was 9.9%, and the profile rate was 56.8%, for a cumulative response rate of 4.0% (4).
